# Evaluation of ^18^F-FMISO PET and ^18^F-FDG PET Scans in Assessing the Therapeutic Response of Patients With Metastatic Colorectal Cancer Treated With Anti-Angiogenic Therapy

**DOI:** 10.3389/fonc.2021.606210

**Published:** 2021-03-17

**Authors:** Sze Ting Lee, Niall Tebbutt, Hui Kong Gan, Zhanqi Liu, John Sachinidis, Kunthi Pathmaraj, Andrew Mark Scott

**Affiliations:** ^1^ Department of Molecular Imaging and Therapy, Austin Health, Melbourne, VIC, Australia; ^2^ Tumor Targeting Laboratory, Olivia Newton-John Cancer Research Institute, Austin Health, Melbourne, VIC, Australia; ^3^ School of Cancer Medicine, La Trobe University, Melbourne, VIC, Australia; ^4^ Department of Surgery, The University of Melbourne, Austin Health, Melbourne, VIC, Australia; ^5^ Department of Medicine, The University of Melbourne, Austin Health, Melbourne, VIC, Australia; ^6^ Department of Medical Oncology, Olivia Newton-John Cancer Research Institute, Melbourne, VIC, Australia

**Keywords:** metastatic colorectal carcinoma, fluoromisonidazole (FMISO) positron emission tomography (PET), hypoxia, bevacizumab, angiogenesis, response

## Abstract

**Introduction:**

Tumor hypoxia and angiogenesis are implicated in tumor growth and metastases, and anti-angiogenic therapies have an important role in treating patients with metastatic colorectal cancer. However, the prevalence of hypoxia has not been fully evaluated in colorectal liver metastases, and hypoxic response to anti-angiogenic therapy has not been clearly established. The aims of the study were to evaluate the changes seen on ^18^F-FMISO and ^18^F-FDG PET scans in patients treated with anti-angiogenic therapy, and to correlate these measures of hypoxia and metabolism with clinical outcomes, and blood biomarkers of angiogenesis.

**Methods:**

Patients with metastatic colorectal carcinoma planned for treatment with bevacizumab and chemotherapy received routine staging investigations prior to any treatment, including a FDG PET scan. A FMISO PET scan was performed within 4 weeks of staging tests, with blood specimens collected at that time for serum VEGF and osteopontin measurement. Follow-up FDG and FMISO scans were performed after 1 cycle of treatment. Results were compared to response (RECIST), progression free survival (PFS), and overall survival (OS).

**Results:**

A total of 15 patients were recruited into this prospective trial, of which 13 patients were evaluable for assessment of treatment follow-up. Baseline FDG uptake was higher than FMISO uptake, and there was a significant decrease in FDG uptake (SUV_max_ and TGV) but not FMISO uptake (SUV_max_ and TNR) after treatment. There was a positive correlation between FDG and FMISO SUV_max_ on both baseline and post-treatment PET scans. Blood biomarkers of serum VEGF and osteopontin were significantly correlated with the FDG and FMISO PET parameters.

**Conclusions:**

This study shows that hypoxia in metastatic colorectal cancer, assessed by FMISO PET, shows minor changes following initial treatment with anti-angiogenic therapy, but is associated with therapeutic response. FDG PET uptake changes (SUV_max_, TLG) are also associated with response to anti-angiogenic therapy. These findings demonstrate the interplay between tumor metabolism and hypoxic regulation following anti-angiogenic treatment of metastatic colorectal cancer.

## Introduction

Colorectal cancer (CRC) is the fourth most common cancer type and the second leading cause of cancer-related death according to the WHO GLOBOCAN (1). More than 50% of patients develop metastatic disease, with the most common visceral metastasis found in the liver (2) with 20% of patients presenting initially presenting with synchronous liver metastasis (3). The last few decades has seen improvement in the early detection of colorectal cancer and therapeutic options for metastatic disease, especially with the use of antibody directed therapies according to the mutation status of the tumor (4), and anti-angiogenic treatments, since colorectal cancers is one of the best studied angiogenesis-dependent solid tumors (5).

Tumor angiogenesis has been shown to be a prerequisite for tumor growth beyond 1–2 mm^3^ (6, 7). Most tumors remain restricted in size until the tumor mass expands and overtakes the rate of internal apoptosis by developing blood vessels. This change is the result of a shift in the net balance of stimulators and inhibitors of angiogenesis within the tumor microenvironment (8). In many cancers, the degree of vascularization is inversely correlated with patient survival, and can predict the development of metastases, and tumor growth.

In metastatic colorectal carcinoma, tumor angiogenesis is a key process in tumor growth and metastasis, and is inextricably linked with tumor hypoxia (9). There are many factors involved in the “angiogenic switch”, which is thought to be triggered when hypoxia around a tumor leads to expression of hypoxia response genes (9). Vascular Endothelial Growth Factor (VEGF) messenger RNA (mRNA) levels are dramatically increased within a few hours of exposing different cell cultures to hypoxia, and return to background levels when normal oxygen supply is resumed (10).

Hypoxia-mediated aggressive tumor behavior and resistance to therapy is mediated by several molecular events which allow the adaptation of tumor cells to hypoxia. Positron Emission Tomography (PET) is a non-invasive imaging modality which can be employed to assess hypoxia. The most validated radiotracer for detection of hypoxia is ^18^F-Fluoromisonidazole (^18^F-FMISO) as it is the only radiotracer that has been validated by multiple studies comparing uptake thresholds to direct pO_2_ measurement with the Eppendorf pO_2_ hypoximeter (11–13). Tumor hypoxia has been implicated in the mechanisms of resistance to chemoradiotherapy in several malignancies, therefore may be the key to the resistance mechanisms of chemoradiotherapy in rectal adenocarcinoma (14).

The presence and impact of hypoxia on patients with metastatic colorectal cancer is not well understood, and imaging and biochemical markers correlating hypoxia and therapy response may have a significant role in the management of colorectal cancer patients, particularly as a predictor of tumor response to chemotherapy with angiogenic inhibitors.

VEGF has also been reported to be relatively higher in colorectal metastases compared to the primary lesions, but may also vary with the location of metastatic disease (15, 16). One study has shown a correlation between the VEGF mRNA of liver metastasis and primary colon tumor tissue (15), and VEGF binding stimulates endothelial cell proliferation, migration and survival (16). The key role angiogenesis plays in cancer growth and metastasis makes it a very attractive target for developing anticancer therapies. Bevacizumab was the first anti-VEGF therapy that was approved for patients with metastatic colorectal carcinoma, with combination treatments of bevacizumab and chemotherapy resulting in prolonged overall survival (17). However, there is a lack of data available on the impact of bevacizumab on hypoxia regulation in metastatic colorectal cancer, especially in the setting of imaging with hypoxic PET tracers. Osteopontin is a secreted multifunctional glycophosphoprotein which is also involved in angiogenesis, and a large meta-analysis of patients with colorectal carcinoma showed that high osteopontin expression was significantly associated with high tumor grades, metastatic disease, with prognostic implications (18).

^18^F-fluorodeoxyglucose (FDG) is widely used to stage and monitor treatment response in metastatic colorectal cancer. FDG uptake depends on the expression of GLUT1, which is over-expressed on tumor cells, but the FDG uptake is dependent on tumor vascularity and might be influenced by tumor hypoxia (19).

The primary aim of this study was to assess changes in hypoxia and glucose metabolism in metastatic colorectal cancer following anti-angiogenic therapy by ^18^F-FMISO and ^18^F-FDG PET scans, and to correlate these findings with patient outcomes. The secondary aim was to assess the correlation between blood biomarkers of angiogenesis (VEGF and osteopontin) and PET-derived parameters of hypoxia and metabolism.

## Methods

Patients with metastatic colorectal carcinoma considered appropriate for treatment with chemotherapy and anti-VEGF inhibitor (bevacizumab) were recruited to this study. All patients had pre-treatment routine staging investigations as deemed appropriate by the treating clinician, in the form of diagnostic CT chest, abdomen, pelvis and ^18^F-FDG PET scan. A subsequent ^18^F-FMISO PET scan was performed within 4 weeks of the FDG PET scan prior to commencement of treatment with pre-treatment bloods collected. The patients were treated with bevacizumab as part of a combined therapy regimen, which was started within 4 weeks of the FMISO PET scan. The dose of bevacizumab was 7.5 mg/kg per dose, given every 3 weeks. Follow-up FDG and FMISO PET scans were performed within 2–3 weeks after the 1^st^ cycle of treatment which included bevacizumab and standard chemotherapy. Routine post-treatment restaging CT scans were performed at regular intervals, and the patients were followed-up clinically for up to 5 years. All patients signed an informed consent form to participate in this institutional ethics approved trial.

The PET scans were performed on a Philips Gemini PET scanner (Philips Medical Systems, Cleveland, OH, USA) which included a low dose 30 mA/slice 2-slice CT for purposes of attenuation correction and anatomical localization. The PET emission scan duration was for 3 min per bed position, and the patients were scanned from skull base to mid-thigh with arms raised above their heads for the FDG PET scan. The FMISO scan was performed of the abdomen and pelvis in most patients, extending to include the lungs in patients with known metastatic lung disease. For the FDG PET scans, the patients were fasted for a minimum of 6 h prior to injection of approximately 5 MBq/kg of ^18^F-FDG after a 60 min uptake time. Patients did not fast for the FMISO PET scans, where they were injected with approximately 370MBq of ^18^F-FMISO intravenously and imaged after a 2 h uptake time.

Both FDG PET and FMISO PET scans were analyed using MedView^®^ (MedImage Inc. Ann Arbor, MI, USA) software platform to assess the SUV_max_ of tumor and normal liver, with a ratio of tumor to normal liver SUV_max_ subsequently calculated. A visual uptake score of the tumor was also performed, rating tumor uptake from a scale of 1–5. A score of 1 had uptake less than normal liver; a score of 2 had uptake similar to normal liver; as score of 3 had mildly increased uptake compared to normal liver; a score of 4 had moderately increased uptake compared to normal liver; and a score of 5 had markedly increased uptake compared to normal liver. Semi-quantitative analysis of the PET scans was performed to assess for the maximum standardised uptake (SUV_max_) in the region of interest on both FDG and FMISO PET scans. The total glycolytic volume (TGV) on the FDG PET scan and tumor to normal ratios (TNR) for the FDG and FMISO PET scan were also obtained. These values were subsequently correlated with the blood biomarkers of VEGF and osteopontin.

### Blood Biomarkers

Correlative blood specimens were also obtained at the time of the FMISO PET scan, and archived in the Victorian Cancer Biobank for further analysis. The blood samples were analyzed for plasma VEGF and osteopontin, biomarkers of angiogenesis and hypoxia, using commercially available ELISA kits (R&D Systems; catalogue numbers QVE00B and DOST00 respectively). The experiments were performed according to the manufacturer’s instructions.

### Statistical Analysis

Statistical analysis was performed using Prism 8 for MacOS^®^ version 8.4.1 (GraphPad Software LLC) statistical software package, with paired t-test, ANOVA, and progression-free survival and overall survival analysis performed as suitable. For the comparison of means, Student t-test was employed where only two groups were being considered. For survival analysis, median values were used to assess the discriminative performance of FDG PET parameters of SUV_max_, TGV or TNR, and FMISO SUV_max_, but the well-established FMISO TNR value of 1.2 was used (11). A p-value of <0.05 was considered statistically significant.

## Results

### Patient Demographics

There were 15 patients (9M:6F) recruited into this study, mean age 56.8 years (range 23 to 77 years). Thirteen patients had evaluable baseline and follow-up PET scans performed. One patient died after one cycle and another patient withdrew from the study after the baseline scans, therefore follow-up scans could not be performed. The patient demographics are summarized in **Table 1**. Most metastatic lesions were in the liver, with the remainder of metastases within intra-abdominal nodes. The average size of the largest metastatic lesions per patient measured at 43mm on pre-treatment CT scan.

**Table 1:** Therapy response cohort patient demographics.

Patient No.	Age	Sex	Primary Tumor	Site of metastasis	Reference lesion size (mm)
1	69	F	Transverse colon	Liver	44
2	75	M	Rectum	Liver Lung	42 13
3	65	M	Descending colon	Liver Lung	98 25
4	77	M	Sigmoid	Para-aortic LN Liver	39 28
5	75	M	Rectum	Liver	62
6	40	M	Sigmoid	Liver (multiple >100)	50
7	64	F	Rectum	Liver	22
8	53	F	Rectosigmoid	Liver	40
9	23	F	Sigmoid	Liver	20
10	50	F	Sigmoid	Lung Para-aortic LN	34 22
11	63	M	Splenic flexure	Liver Lung	45 29
12	70	M	Caecum	Mesenteric mass Retroperitoneal LN	40 25
13	50	F	Ascending colon	Omentum Liver	32 21
14	27	M	Rectal	Para-aortic LN	27
15	51	M	Ascending colon	Liver	22

### Positron Emission Tomography Scan Assessment

**Figure 1:**
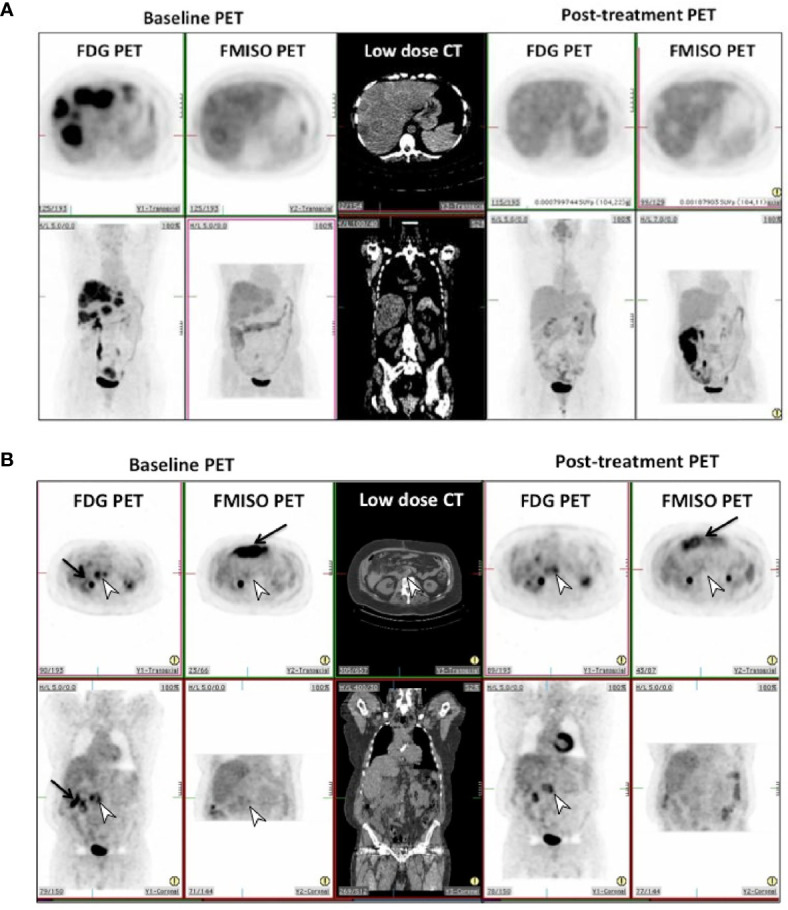
**(A)** Pre and post-treatment FDG and FMISO PET scans in patient 8 in transverse (top row) and coronal (bottom row) projections. This shows complete metabolic and hypoxic response in the liver metastases to treatment, with photopenic defects in the liver on post-treatment scans. **(B)** Pre and post-treatment FDG and FMISO PET scans in patient 12 in transverse (top row) and coronal (bottom row) projections. This shows incomplete metabolic and hypoxic response to treatment on FDG & FMISO PET in retroperitoneal lymph nodes seen on CT (white arrowhead). Intense bowel activity on FMISO scans is noted anteriorly (black arrows).

On visual assessment of baseline PET scans, all patients had a more intense FDG uptake in the reference lesion(s) compared to FMISO uptake, as summarized in **Table 2**. On the follow-up PET scans, the FDG PET did visually decrease by at least 1 point, but on FMISO scans, the visual scores either remained the same or only decreased by 1 point in all patients, apart from one patient who initially scored a 4 and this decreased to 1 on their follow-up. **Figures 1A, B** show FDG and FMISO changes in liver metastases and extrahepatic metastases respectively.

**Table 2:** PET scan findings and therapy response.

Pt. No.	FDG PET								FMISO PET							
	Baseline			Follow-up			SUVmax Response	TGV Response	Baseline			Follow-up			SUVmax Response	TNR response
	Visual	SUVmax	TGV	Visual	SUVmax	TGV			Visual	SUVmax	TNR	Visual	SUVmax	TNR		
1*	5	8.1	603.15	N/A	N/A	N/A	N/A	N/A	3	2.1	1.0	N/A	N/A	N/A	N/A	N/A
2	5	5.4	7,777.3	4	3.6	777.73	-33%	-90%	2	2.7	1.1	2	2.7	1.2	+0.4	+3%
3	5	10.6	382.27	5	8.4	164.38	-21%	-57%	4	3.6	1.4	3	2.8	1.2	-22%	-15%
4	4	4.3	131.91	3	2.7	6.60	-37%	-96%	2	2.1	1.0	2	2.0	0.9	-5%	-13%
5*	5	9.1	258.77	N/A	N/A	N/A	N/A	N/A	1	2.0	1.0	N/A	N/A	N/A	N/A	N/A
6	5	7.0	3,951.7	3	2.5	237.10	-64%	-94%	3	2.5	1.3	2	2.3	1.1	-8%	-20%
7	4	4.3	70.92	3	1.9	24.82	-56%	-65%	3	2.1	1.2	2	2.0	1.1	-5%	-5%
8	5	8.7	3,749.0	4	3.2	674.82	-63%	-82%	4	3.8	1.5	1	3.0	0.9	-16%	-42%
9	5	4.7	18.13	3	1.7	0.90	-64%	-95%	2	1.7	1.0	2	1.6	1.0	-6%	-6%
10	5	5.7	369.80	4	3.7	214.48	-35%	-42%	2	2.2	1.8	2	2.1	1.1	-5%	-37%
11	5	6.1	612.78	5	4.1	428.95	-33%	-30%	2	2.6	1.1	2	2.4	1.0	-8%	-8%
12	5	6.1	351.76	5	5.2	157.85	-15%	-55%	3	3.2	1.2	2	2.6	0.9	-19%	-25%
13	5	15.2	4,016.9	5	10.9	2811.9	-28%	-30%	3	3.8	1.2	2	3.5	1.2	-8%	+2%
14	5	3.7	283.91	5	3.6	332.17	-2%	17%	2	1.7	1.2	2	1.2	1.6	-29%	+33%
15	5	9.1	283.99	5	6.0	225.35	-34%	-21%	2	2.2	1.0	2	2.2	1.0	0	0
**Mean +/- SD**		**7.2 +/- 3**	**1,524 +/- 2,281**		**4.4+/- 2.7**	**466+/- 744**	**-37% +/- 20**	**-57+/- 35**		**2.6 +/- 0.72**	**1.2 +/- 0/23**		**2.3 +/- 0.6**	**1.1 +/- 0.19**	**-10 +/- 8.8**	**-10 +/- 19**

*Patient 1 died after treatment, before post-treatment PET; *Patient 5 withdrew from study after baseline PET scans.

TGV, Total Glycolytic Volume; TNR, Tumor to Normal Ratio.

The SUV_max_ for baseline FDG uptake in tumor was higher compared to SUV_max_ for FMISO (**Table 2**). There was a statistically significant association of all baseline PET parameters (FDG SUV_max_, FMISO SUV_max_, FDG TGV and FMISO TNR) using ANOVA analysis, with a p-value of 0.02. Following anti-angiogenesis therapy, the average decrease of mean SUV_max_ on FDG PET was 4.58 (range 1.7-10.9) on the follow-up scans available (n=13). The mean difference in follow-up FDG SUV_max_ was a reduction of 2.79 +/- 1.11 (p-value 0.02). For the FMISO PET scans, the baseline FMISO SUV_max_ was 2.55 (range 1.7–3.8), with a decrease in the mean SUV_max_ to 2.36 (range 1.2–3.5) on the follow-up scans available. The mean decrease in follow-up FMISO SUV_max_ was 0.28 +/- 0.3, with a non-significant p-value of 0.36.

The tumor to normal ratio (TNR) on baseline FDG PET had a mean of 3.4 +/- 0.42 (range 1.6–6.2), with an average decrease in mean TNR to 2.4 +/- 0.5 on the follow-up scans (**Table 2**). The mean difference in FDG TNR was a reduction of 1.01 +/- 0.59 (p-value 0.11). The FMISO TNR had a mean of 1.2 +/- 0.08 on baseline (range 0.9–2.1), with a decrease in the mean TNR to 1.12 +/- 0.06 (range 0.9–1.6) on follow-up scans. The mean decrease in FMISO TNR was 0.09 +/- 0.1, with a non-significant p-value of 0.39.

The total glycolytic volume (TGV) on baseline FDG PET scans was 1693 +/- 669.9 (range 18.3–7,777 SUV*ml), whilst the mean TGV on follow-up FDG PET scans was 465.9 +/- 206.4 (range 0.9–2,812 SUV*ml). The mean decrease in TGV was -1227 +/- 590 SUV*ml, with a significant p-value of 0.012.

**Figure 2:**
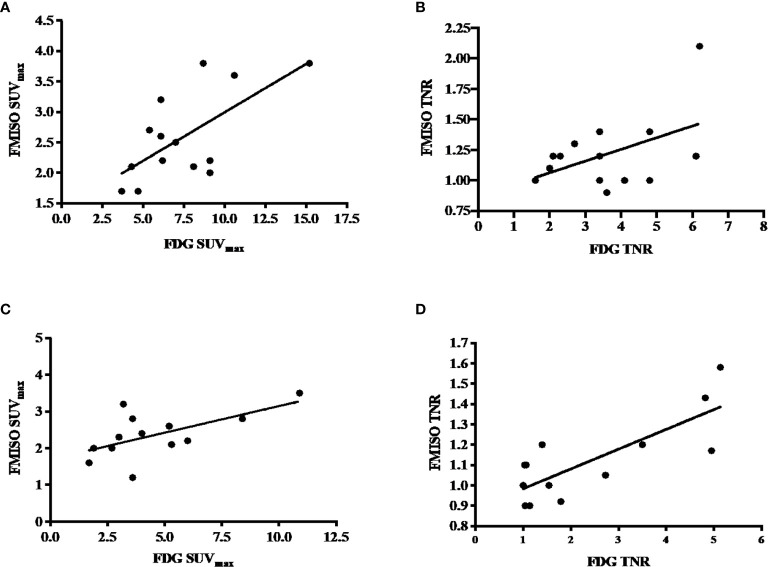
**(A)** Correlation between baseline FDG and FMISO SUV_max_, with a Pearson’s correlation of 0.67 and p-value of 0.007. **(B)** Correlation between baseline FDG and FMISO TNR, with a Pearson’s correlation of 0.49 with a non-statistically significant p-value of 0.06. **(C)** Correlation between post-treatment FDG and FMISO SUVmax, with a Pearson’s correlation of 0.61 and p-value of 0.06. **(D)** Correlation between post-treatment FDG and FMISO TNR, with a Pearson’s correlation of 0.79 and p-value of 0.0014.

There was a positive correlation between FDG and FMISO uptake as measured by SUV_max_ on both baseline and follow-up PET scans. On the baseline PET scans, there was a statistically significant correlation between the SUV_max_ on the FDG and FMISO PET, with a Pearson’s r-value of 0.67 (p-value 0.007), but not the baseline tumor to normal ratio (TNR), which had a Pearson’s r-value of 0.49 (p-value 0.06), these are shown in **Figures 2A, B**. On the follow-up scans, there was a statistically significant correlation between the SUV_max_ of the FDG and FMISO PET scans with a Pearson’s r-value of 0.61 (p-value 0.027), as well as the FDG and FMISO TNR, with a Pearson’s r-value of 0.79 (p-value 0.0014) as shown in **Figures 2C, D**.

### Treatment Response Assessment

**Figure 3:**
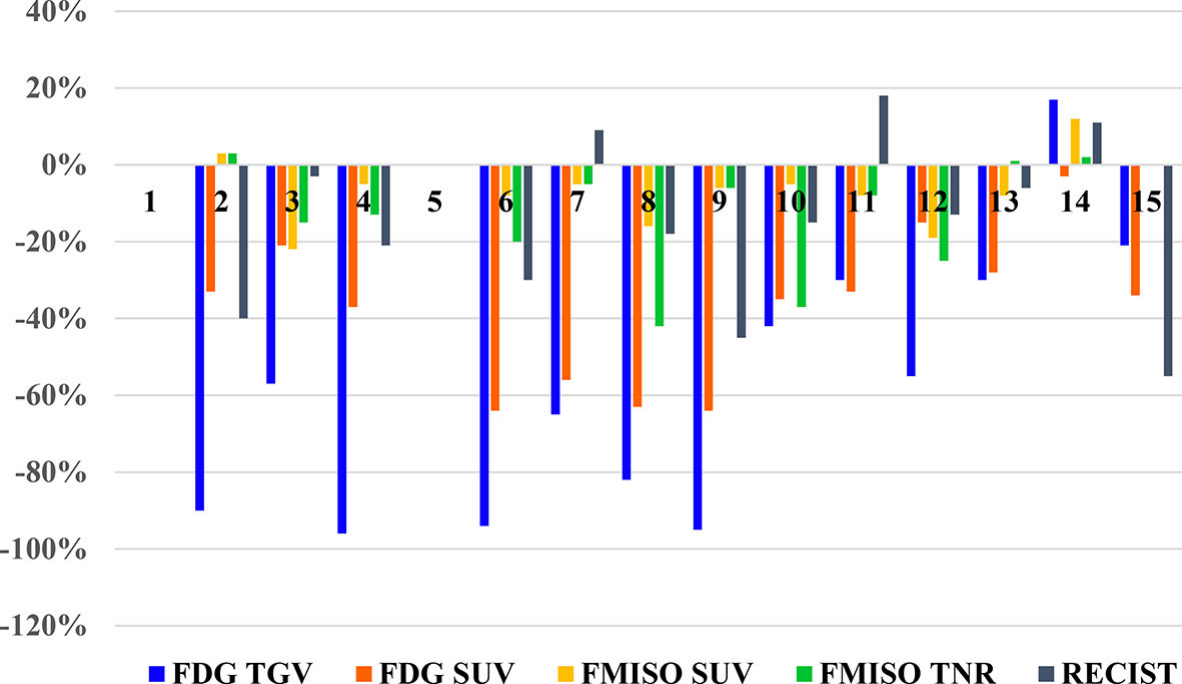
Waterfall plot of the changes seen of PET parameters on FDG and FMISO PET scans, and RECIST measurements on diagnostic CT for each patient.

There were 13 patients with evaluable treatment response, as two patients (Patient 1 and 5) did not have follow-up scans performed. These results are illustrated in the waterfall plot compared to anatomic assessment of response by with CT scan (RECIST), as seen in **Figure 3**.

According to the RECIST 1.1 criteria for evaluation of treatment response on CT scan, there were four patients with partial response with a decrease in tumor lesion size of at least 30% from the reference, and nine patients had stable disease, with insufficient shrinkage to qualify for partial response and insufficient increase to qualify for progressive disease (**Table 3**).

**Table 3:** Diagnostic CT findings and therapy response.

Pt. No.	Baseline lesion size (mm)	Follow-up lesion size (mm)	Response in lesion size (RECIST)	Tumor volume (mm^3^)
1*	44	N/A	N/A	119.55
2	42	25	-40%	1,446.78
3	98	95	-3%	127.62
4	39	31	-21%	44.86
5*	62	N/A	N/A	55.84
6	50	35	-30%	970.62
7	22	24	9%	19.39
8	40	33	-18%	744.19
9	20	11	-45%	6.91
10	34	29	-15%	112.9
11	45	53	18%	195.71
12	40	35	-13%	82.82
13	32	30	-6%	919.68
14	27	30	11%	94.72
15	22	10	-55%	75.71
**Mean** **+/- SD**	**41.13 +/- 19.4**	**33.92 +/- 21.3**	**-16% +/- 22.3**	**334.5 +/- 452.4**

*Patient 1 died after treatment, before post-treatment PET.

*Patient 5 withdrew from study after baseline PET scans.

Using the EORTC/NCI criteria for evaluation of metabolic response on FDG PET scan, there were 12 patients with partial metabolic response (decrease in SUV_max_ of 15%–25%), of which 10 patients had a decrease of >25%, and 1 patient had stable metabolic disease with a small decrease in SUV_max_ of 2% (**Table 2**). Although similar criteria could not be applied to FMISO uptake in response to treatment, there were two patients with a decrease in FMISO SUV_max_ of >20%, two patients with moderate decrease in FMISO SUV_max_ between 10%–20%, seven patients who had small decreases in FMISO SUV_max_ of <10%. There were two patients with no change in FMISO uptake.

The TGV changes on FDG PET following treatment were greater, with eight patients demonstrating decreases in TGV of >50%; while four patients had decreases in TGV between 20%–50%, and one patient had an increase of 17% (**Table 2**). The tumor to normal ratio (TNR) changes on FMISO PET showed four patients had decreases between 20%–50%; two patients had decreases between 10%–20%; three patients had minor decrease of <10%; one patient had no change in FMISO TNR; and two patients had minor increase of up to 10%. One patient had an increase of 33%, who was also the single patient who demonstrated an increase in burden of disease on FDG PET too.

### Blood Biomarkers and Positron Emission Tomography Scan Parameters

To evaluate the PET scan parameters of TGV and SUV_max_ for FDG PET scans, and TNR and SUV_max_ for FMISO PET scans, compared to the blood biomarker of VEGF and osteopontin, one-way ANOVA was performed. The ANOVA analysis indicated that there was a statistically significant correlation between all the PET parameters and VEGF levels and osteopontin levels in each patient (p-value <0.05).

### Survival Assessment

**Figure 4:**
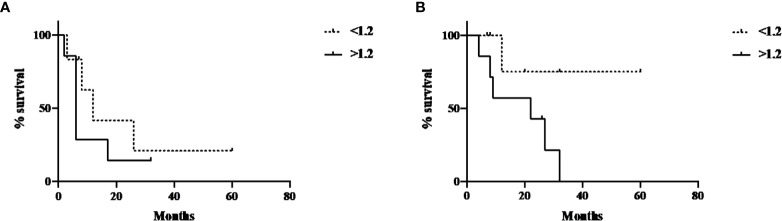
Post-treatment FMISO TNR and PFS and OS. **(A)** Post-treatment FMISO TNR and PFS (p-value 0.16); **(B)** Post-treatment FMISO TNR and OS (p-value 0.14).

Progression-free survival and overall survival in this cohort of patients was also evaluated, using a median cut-off of 6.6 for FDG SUV_max_, 2.25 for FMISO SUV_max_, 333 for FDG TGV. A cut-off value of 1.2 was used for FMISO TNR according to previously well published data (11). These were not statistically significant for neither progression-free or overall survival for all four PET parameters. However, the survival curves for the post-treatment FMISO TNR did demonstrate a trend towards significance, with p-value of 0.16 for PFS and 0.14 for OS (**Figures 4A, B**).

## Discussion

Tumor hypoxia and angiogenesis are hallmarks of cancer, and tumor cells have been found to be accompanied by angiogenic blood vasculature that exhibited altered permeability and vessel compression, with compressed and non-functional lymphatic vessels (20). These features lead to increased interstitial fluid pressure, which is a major transport barrier for the delivery of chemotherapeutic agents in tumor (20, 21). The concept of vascular “normalization” by anti-VEGF agents for improved drug delivery and efficacy is the basis of anti-angiogenic treatments (e.g., bevacizumab) which has been approved for treatment of metastatic colorectal cancer.

Our study evaluating FMISO response in patients with metastatic colorectal carcinoma treated with bevacizumab has demonstrated that hypoxia is present at baseline, but there is only minor changes in hypoxia in the tumor following initial treatment. While FDG uptake in the metastases was greater than FMISO uptake at baseline, FDG change in response to treatment was also greater than hypoxia change. Changes in FDG uptake (particularly TGV) were also associated with the response to anti-angiogenic treatment. These findings indicate that there is a complex interplay between tumor metabolism and hypoxic levels following anti-angiogenic treatment of metastatic colorectal cancer.

Following treatment with bevacizumab, changes in FMISO TNR showed a correlation with progression free and overall survival, although this did not reach statistical significance. To our knowledge, our study is the first to explore dynamic changes in hypoxia following bevacizumab treatment in colorectal cancer patients. In a prior study, a preclinical model of colorectal xenografts treated with bevacizumab demonstrated that post-treatment FMISO scan demonstrated a trend toward lower FMISO uptake, however, this did not correspond to hypoxia related parameters of carbonic anhydrase IX and GLUT1 staining which increased following treatment. This is likely due to the “normalization” of tumor vasculature and permeability following bevacizumab which improves blood supply to the tumor (22, 23). Changes in vascular morphology following anti-angiogenesis therapy have been reported from a study of patients with hepatic liver metastases where treatment with bevacizumab resulted in tumor vessel stabilization, reduced vascular density and increased necrosis (24).

The baseline VEGF and osteopontin levels correlated significantly with all the baseline PET parameters on FDG and FMISO scans, indicating that the serum levels of these markers are reflective of the underlying level of hypoxia in patients with metastatic colorectal cancer.

The study limitations are this is a pilot study, and there were a small number of patients who had PET scans available for comparison post-treatment. The changes in vascularization, and hence changes in hypoxia with anti-angiogenic treatments may also require a longer period of treatment before significant changes in hypoxia level are seen to be able to be assessed by FMISO PET scans, which were performed after 1 cycle of treatment in our study. In addition, possible changes in normal liver blood flow following anti-angiogenic therapy and subsequent impact on FMISO uptake and TNR values are not fully understood, and could be addressed in future studies with direct measurement of normal liver blood flow changes following anti-angiogenic therapy.

This study has shown that hypoxia in metastatic colorectal cancer, assessed by FMISO PET, is present and shows minor changes following initial treatment with anti-angiogenic therapy, but is associated with therapeutic response. FDG PET uptake changes (SUV_max_ and TGV) are also associated with response to anti-angiogenic therapy. Both FDG and FMISO PET parameters showed significant correlation with biomarkers of angiogenesis. These findings demonstrate the complex interplay between tumor metabolism and hypoxic regulation following anti-angiogenic treatment of metastatic colorectal cancer.

## Data Availability Statement

The original contributions presented in the study are included in the article/**Supplementary Material**. Further inquiries can be directed to the corresponding author.

## Ethics Statement

The studies involving human participants were reviewed and approved by the Austin Health Human Research Committee. The patients/participants provided their written informed consent to participate in this study.

## Author Contributions

SL made the patient review, consent, experimental design and preparation, manuscript preparation, PET review, and image analysis. NT made the patient review, experimental design, consent, and manuscript review. HG made the experimental design, statistical analysis, and manuscript review. ZL performed the laboratory experiments and gave advice. JS made the PET tracer preparation and QC. KP. made the PET scan acquisition and QC. AS made the experimental design, manuscript preparation and review, and PET review. All authors contributed to the article and approved the submitted version.

## Funding

Funding from the Cancer Council Victoria and University of Melbourne for STL, NHMRC grants (Nos. 1084178 and 1092788), and Operational Infrastructure Support Program provided by the Victorian Government is acknowledged. AS is supported by an NHMRC Investigator Fellowship.

## Conflict of Interest

The authors declare that the research was conducted in the absence of any commercial or financial relationships that could be construed as a potential conflict of interest.
