# Supplementary material for: Evaluation of 18F-FMISO PET and 18F-FDG PET Scans in Assessing the Therapeutic Response of Patients With Metastatic Colorectal Cancer Treated With Anti-Angiogenic Therapy
Source: Front Oncol. 2021 Mar 17;11:606210. doi: 10.3389/fonc.2021.606210 (PMC8010243; doi:10.3389/fonc.2021.606210)
Supplement: Supplementary file 1 [file DataSheet_1.docx]

**Appendix 1: Progression Free Survival and all PET parameters**

PET parameters and PFS: A) FDG SUV_max_ (p-value 0.74); b) FMISO SUV_max_ (p-value 0.8); C) FDG TGV (p-value 0.97); D) FMISO TNR (p-value 0.16)

**A**

**B**

**C**

**D**

**Appendix 2: Overall Survival and all PET parameters**

A) FDG SUV_max_ (p-value 0.70); B) FMISO SUV_max_ (p-value 0.77); C) FDG TGV (p-value 0.84); D) FMISO TNR (p-value 0.14)

**A**

**B**

**C**

**D**
